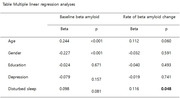# Relationship between disturbed sleep and longitudinal changes in cerebral beta‐amyloid accumulation in cognitively normal elderly adults

**DOI:** 10.1002/alz.089892

**Published:** 2025-01-03

**Authors:** Min Ji Lee, Musung Keum, Jee Wook Kim, Guk‐Hee Suh, Young Min Choe, Alzheimer’s Disease Neuroimaging Initiative

**Affiliations:** ^1^ Hallym University Dongtan Sacred Heart Hospital, Hwaseong Korea, Republic of (South); ^2^ Hallym University College of Medicine, Chuncheon Korea, Republic of (South)

## Abstract

**Background:**

In our previous study, using cross‐sectional data, we noted that poor sleep quality during midlife increases pathological beta‐amyloid protein (Aβ) deposition. This study aimed to investigate the relationship of disturbed sleep and longitudinal changes in cerebral Aβ accumulation in elderly individuals with normal cognition.

**Method:**

Three‐hundred and two cognitively normal old adults from the Alzheimer’s Disease Neuroimaging Initiative received (^18^F)‐florbetapir positron emission tomography (PET) scans. Sleep disturbance and depression were measured using the baseline Neuropsychiatric Inventory (NPI) or NPI‐Questionnaire sleep and depression items. Multiple linear regression analysis was performed to examine the relationship between poor sleep and baseline Aβ deposition and rate of change in Aβ accumulation after adjusting for age, gender, education, and depression.

**Result:**

In cognitively normal elderly individuals, poorer sleep was significantly associated with higher rates of Aβ accumulation after controlling for age, gender, education and depression. In contrast, we could not find any significant relationship between sleep disturbance and baseline Aβ deposition.

**Conclusion:**

Our results suggest that disturbed sleep accelerates longitudinal Aβ accumulation in cognitively healthy old adults. On the other hand, the present results do not support the hypothesis of opposite direction that Aβ deposition influences sleep disturbance.